# Sex differences in allostatic load trajectories among midlife and older adults: Evidence from the China health and retirement longitudinal study

**DOI:** 10.1371/journal.pone.0315594

**Published:** 2024-12-26

**Authors:** Guannan Li, Gindo Tampubolon, Asri Maharani, Chenglin Tu

**Affiliations:** 1 Global Development Institute, School of Environment, Education and Development, The University of Manchester, Manchester, United Kingdom; 2 Division of Nursing, Midwifery and Social Work, School of Health Sciences, Faculty of Biology, Medicine and Health, The University of Manchester, Manchester, United Kingdom; 3 Guangzhou Development Research Institute, Guangzhou University, Guangzhou, China; Dynamical Business & Science Society - DBSS International SAS, COLOMBIA

## Abstract

The female advantage in life expectancy sits uneasily with female disadvantage in health and well-being in later life compared to their male counterparts. This health disparity has been suggested to rest on sex difference in allostatic load (AL). We aim to delineate the sex-specific age trajectories of AL among midlife and older adults in China and to interpret the contradiction between the female advantage in life expectancy and their disadvantage in health in later life from the perspective of physiological dysregulation. Using data from the China Health and Retirement Longitudinal Study (CHARLS) conducted in 2011 and 2015, we included 3,836 male and 3,308 female Chinese adults aged 45 and older. Two-level mixed-effects models were fitted to examine how AL changed over time. Missing values were addressed by performing multiple imputations using chained equations. Results show AL increases with age for both sexes, with a steeper rise in females and a slight decline in males after adjusting for the sex-age interaction. Older males born before the People’s Republic of China (PRC) exhibited different AL trajectories from younger cohorts. The sex-specific trajectories converge around the late 60s, with females surpassing males, aligning with the life expectancy-health paradox. The presence of a healthier older male cohort in CHARLS suggests future studies should account for cohort effects.

## Introduction

Available evidence confirms that life expectancy differs at all ages, with females living longer than males [1, 2]. However, the sex gap in life expectancy converges with age [3]. Research indicates that, despite their longevity, females experience a decline in quality of life, poorer health status and higher morbidity, compared to their male counterparts in later life [4]. Specifically, older females report a higher incidence of physical disabilities [5, 6], a greater prevalence of falls, and lower performance on balance and walking tasks [7–11]. Studies also find that older females tend to have greater adiposity and less lean mass, partly due to lower habitual physical activity and cardiorespiratory fitness [12–14]. Beyond differences in physical activity, body composition and aerobic fitness, the literature further suggests that differences in physiological dysregulation, particularly allostasis and allostatic load, also play a crucial role in explaining these health disparities between males and females in later life [3, 15].

Allostasis refers to the body’s ability to achieve stability through change, involving mediators like those from the immune system, autonomic nervous system (ANS), and hypothalamo-pituitary-adrenal (HPA) axis. However, chronic overactivation of these systems can lead to allostatic load (AL), which is the wear and tear on the body and brain, manifesting as conditions such as accumulation of abdominal fat, loss of bone minerals, and hippocampal nerve cell atrophy [16]. Hippocampus exhibits significant structural plasticity influenced by circulating hormones and stress. However, repeated stress can remodel dendrites, suppress neurogenesis, and regulate synapse formation [16, 17]. Such effects are particularly evident in female rats [18]. Translating these findings to humans is challenging due to a lack of similar studies on the human brain. However, research indicates that postmenopausal women are more susceptible to declines in hippocampal-dependent cognitive functions, which are associated with higher levels of urinary cortisol, a steroid hormone produced by the adrenal glands in response to stress [19]. Despite this vulnerability, the female brain shows better resilience compared to males, through neurogenesis, dendritic remodeling, and synaptic plasticity, with estrogen playing a role in maintaining this resilience. Estrogen, which is primarily produced in the ovaries, have been identified as potential neuroprotective agents that could mitigate the effects of AL [16]. While AL, has been increasingly used as a broad measure of the cumulative burden of physiological dysregulation [20, 21] and as a comprehensive index of population frailty, capable of predicting major health outcomes and mortality in older age groups [22, 23].

Recent population-based studies have indicated significant sex differences in several biomarkers that are related to AL [24–32], such as in C-reactive protein [25, 27–29], interleukin-6 [28] and many other chronic disease-related biological risk factors [30]. However, these studies often focus on individual rather than overall physiological burden and are typically restricted to small, homogeneous samples in terms of health status [33], demographics [34], or geography. The complexity of age variations in sex differences in biological functions remains largely unexplored and inadequately measured in population-based samples. Further, due to the high cost of collecting and storing biological data, most studies are conducted in developed countries.

Among the few research, there is still a notable gap in the literature concerning how sex differences in AL as individuals age. Yang and Kozloski’s [15] cross-sectional study on Americans reveals that, given their higher morbidity rates, females exhibit higher AL levels than males, indicating greater physiological dysregulation. This finding presents a paradox: a higher level of physiological dysregulation in females appears to contradict their longer life expectancy. To response to this paradox, Tampubolon and Maharani [3] delineate the sex-specific age trajectories of AL among older Americans and Britons, respectively. Using a longitudinal study design, they find that despite AL trajectories for both sexes converging with age, females generally have lower AL levels than their male counterparts in both countries.

The contradictory differences between female longevity and their poorer health in later life are also pronounced in China, one of the largest developing countries in the world. It has been projected that by 2030, the life expectancy at birth for Chinese females will be 79 years, compared to 76 years for males [35]. However, despite higher life expectancy, older Chinese females report higher morbidity due to conditions such as hypertension, diabetes, angina, arthritis, suicide, depression, and dementia [35].

The study of AL has been increasing in China, driven by the availability of new biomarker datasets. However, most research interests are focused on exploring the risk factors for allostatic load from the perspective of socio-economics [34, 36–38]. There is a gap in knowledge concerning the sex differences in AL and the contradiction between female advantages in longevity and their disadvantage in health outcomes, as explained from the perspective of physiological dysregulation. Therefore, in the present study, we aim to address several key questions regarding AL among midlife and older adults in China:

First, we seek to determine if there is a sex difference in the age trajectories of AL. Second, given the female advantage in life expectancy, we investigate whether there is a female advantage [3] or a male advantage [15] in AL. Third, since most relevant studies have been conducted in developed countries, we aim to explore if studying China, one of the largest developing countries, can enhance our understanding of AL trajectories and physiological dysregulation in a heterogeneous cultural and socioeconomic context distinct from the West. Last, by controlling for a set of covariates, we examine the extent to which demographic, socioeconomic, and behavioural health factors influence the sex-specific trajectories of AL in middle-aged and older Chinese adults.

## Materials and methods

This study applied the STandardized Reporting Of Secondary data Analyses (STROSA) framework to conduct a secondary analysis of panel data from the China Health and Retirement Longitudinal Study (CHARLS), utilising sociodemographic data from wave 1 (2011) and wave 3 (2015), along with corresponding biomarker data. Please refer to the S1 Checklist in the *Supporting Information*.

### Data source and study sample

We used data from CHARLS, which is a nationally representative longitudinal survey that collects comprehensive data on the health, economic status, and social factors affecting Chinese residents aged 45 and older. Initiated in 2011, CHARLS aims to provide insights into the aging process in China, examining a wide range of variables, including physical and mental health, healthcare access, income, employment, and family dynamics. Blood samples have been only collected in the 2011 baseline survey (11,847 participants) and 2015 survey (13,420 participants), therefore data from these two surveys were used in the present study. As a secondary data, all information from CHARLS has been gathered, transferred, stored and primarily processed by Peking University. More details of CHARLS can be found elsewhere [39, 40].

We excluded participants for whom there was no follow-up (N = 4,199, 35.44%) after the first survey and those who were only sampled in the 2015 survey (N = 5,772, 43.01%). What remained was a balanced longitudinal dataset of 7,648 participants. We excluded 49 (1.05%) participants who reported having a different sex between the two surveys. We also identified an inconsistency in the ages recorded by CHARLS. The period between the two surveys is 4 years, we excluded 259 (3.44%) participants who reported an age gap of more than 6 years between the two surveys. We tolerated a ± 2 years error due to the use of the Chinese lunar calendar or errors in data capture by the project administrators. There were 7,144 participants or 14,288 repeat observations included in the final analysis. Compared to the baseline data, the balanced dataset is slightly older (mean age: 60.60 vs. 59.13), has slightly more females (54.54% vs. 53.52%) and has almost the same allostatic load mean scores (1.199 vs. 1.204). The age range of participants in the final sample is from 45 to 93 years old.

### Ethical considerations

The CHARLS dataset is open-source and accessible to researchers worldwide. Ethical approval for all the CHARLS waves was granted from the Institutional Review Board at Peking University. The IRB approval number for the main household survey, including anthropometrics, is IRB00001052-11015; the IRB approval number for biomarker collection, is IRB00001052-11014.

### Evaluation of allostatic load

We followed previous studies [3, 41] by computing allostatic load score in CHARLS using 10 indicators. The indicators cover four organ systems, including cardiovascular (diastolic blood pressure, systolic blood pressure), inflammation (CRP), metabolic (haemoglobin A1c, high-density lipoprotein/total cholesterol ratio, triglycerides, and fasting glucose), and body fat (BMI and waist circumference). The threshold of the high-risk quartile is replaced with a clinical cut-off when there is one. Such an approach enhances the ability to identify physiological risks. Following literature [42], clinical cut-off points were introduced for other measures (diastolic blood pressure ≥ 90 mmHg, systolic blood pressure ≥ 140mmHg, body mass index (BMI) ≥ 30 kg/m^2^ and a waist circumference of ≥ 102 cm for males and ≥ 88 cm for females). Each biomarker was categorised into 1 (higher than normal values) or 0 (normal values or below). Higher AL scores indicate higher multi-system physiological dysregulation. Further, within each organ system each indicator receives the same proportion. For example, the metabolic system has four biomarkers, each potentially contributing a quarter point when the biomarker value is found in the high risk quartile (zero otherwise) [41, 43]. By following these methods, we are able to compare our results with those from studies conducted in the US and the UK [3].

We also considered how the use of medication would change AL [3]. For participants who were using medication for hypertension or were diagnosed with cardiovascular diseases (CVD) by a doctor, we coded their systolic and diastolic blood pressure readings into 1 to indicate health risk. For participants who were using medication for diabetes or diagnosed with diabetes, we coded their glucose values into 1. And for those using diabetes medication or insulin, their HbA1c values were coded into 1. Existing literature also suggests that diabetic, cholesterol and blood pressure-lowering medication reduced the values of CRP between 25–30%. Therefore, for participants who are using hypertension or diabetes medications or insulin, we coded their CRP values into 1 to indicate health risk if the values are in the second highest 25th percentile [44].

Further, to provide an alternative approach for constructing the AL index and to assess the robustness of the study, we calculated the z-score for each biomarker, following the method outlined by Stephan et al. [3, 45]. Details will be discussed later in this section.

### Covariates

Previous studies have shown that older people’s health varies by income, sex, education, and residence affecting life expectancy and functional limitations among older Chinese [15, 46, 47]. Research has also found individual lifestyles also influence AL [3]. Therefore, factors such as alcohol use, smoking, physical activity, and comorbidities were included to understand their impact on the physiological burden of older Chinese. Specification on survey instruments is listed in *S1 Table*.

### Statistical strategies

#### Bivariate and multivariate analysis

We described the characteristics of respondents at baseline and calculated the mean score of AL by socioeconomic and health behaviour groups. We used Pearson’s *χ*^2^ to compare the level of AL between older males and females in China.

To model changes in AL over time and promote comparability with a previous study [3], we use two-level random-intercept models with time nested within individuals [48]. The model is used for the continuous response variable y_ti_, AL, including age, sex and various other variables as explanatory variables or covariates. The model for the AL y_ti_ of time, _t_, of individual, _i_, is specified as:

$$y_{ti}=\beta_{1}+\beta_{2}x_{2ti}+\cdots +\beta_{p}x_{pti}+\xi_{ti}$$

where x_2ti_ through x_pti_ are covariates and ξ_ti_ is a residual. We assume that the AL of time to individual are uncorrelated given the observed covariates, or in other words that the residuals ξ_ti_ and ξ_t’i_ are uncorrelated. We can therefore split the total residual or error into two error components: ζ_i_, which is shared between time of the same individual, and ǫ_ti_, which is unique for each time:

$$\xi_{ti}=\zeta_{i}+\varrho_{ti}$$

Substituting for ξ_ti_ into the multiple-regression model (1), we obtain a linear random-intercept model with covariates:

$$y_{ti}=\beta_{1}+\beta_{2}x_{2ti}+\cdots +\beta_{p}x_{pti}+\left(\zeta_{i}+\varrho_{ti}\right)$$

$$=\left(\beta_{1}+\zeta_{i}\right)+\beta_{2}x_{2ti}+\cdots +\beta_{p}x_{pti}++\varrho_{ti}$$

This model can be viewed as a regression model with an added level-2 residual *ζ*_*i*_, or with an individual-specific intercept *β*_*1*_ *+ ζ*_*i*_. The random intercept *ζ*_*i*_ can be considered a latent variable that is not estimated along with the fixed parameters *β*_*1*_ through *β*_*p*_. The linear random-intercept model with covariates is an example of a linear mixed (effects) model where there are both fixed and random effects. Such a model is also known as a mixed-effect model.

The models were applied to illustrate the changes in AL and to determine the extent to which different socioeconomic and behavioural health factors contributed to the levels of AL among the older Chinese over time. We then predicted AL scores based on our models and drew the linear trajectories of AL. All analyses were carried out in Stata 15.

#### Attritions and missing values

Missing data on AL (19.00%, N = 1455) and all covariates were handled by multiple imputations (MI) by chained equations [49], conducted by Stata program (Stata, 2009). We imputed missing continuous variables and categorical variables by applying a predictive mean matching imputation method and a multinomial logistic model, respectively. Five cycles of MI were used. We then used the mi estimation function (Stata, 2009) to estimate AL scores by applying the multilevel mixed-effects linear regression and depicting the trajectories accordingly.

#### Sensitivity and robustness analysis

We have carried out four supplementary analyses to examine the sensitivity and robustness of our findings.

Firstly, following Stephan et al. [3, 45], we calculated the z-score for each biomarker and then, we computed the average of those z-scores and produced the final AL indices. This AL indices include nine biomarkers across four organ systems. They are diastolic and systolic blood pressure, CRP, HbA1c, HDL lipoprotein/total cholesterol ratio, triglycerides, fasting glucose, BMI and waist circumference.

Secondly, we examined the potential cohort effects on the trajectories of AL. It has been documented that early-life experiences could influence individuals’ health and well-being in later life [50]. Thus, it is necessary to consider the political system, cultural and societal changes when studying the health and well-being of older people in China [51]. Chinese people who were born before the 1950s (the People’s Republic of China (PRC) was found in 1949) suffered during World War II and civil wars, leading to tremendous stress and malnutrition in their early life. While people who were born later, benefited from a relatively stable and much less stressful environment, improved economy and health infrastructures, which significantly improved their health and quality of life. We, thus, posited that there are age cohorts that have different AL trajectories. To examine the cohort effects, we regrouped participants into two categories based on their year of birth: people who were born before 1950 and those who were born in 1950 and later. Following the same procedures as described above, we set out to draw the sex-specific trajectories of AL for each cohort.

Because the repeated observations have shrunk due to attrition, we follow the extensive literature in using inverse probability weights (IPW) [52–54]. In our third supplementary analysis, the IPW were computed with the logistic model including age, sex, marital status, residence, education, wealth and cognition level (measured by episodic memory). Following the same procedures described above (without MI), we added these weights to a new random-intercept model to compare to the original one. Further, we added weights to models responding to different age cohorts and weights to the model in which AL is defined by z-score, respectively.

In general, mixed-effect models can handle unbalanced data, measuring changes in a certain variable over time. However, three waves are preferred for robust estimations [55]. In a two-wave longitudinal study, using a balanced dataset ensures enhanced comparability across time points, reduces bias, and improves statistical power. This approach allows for consistent tracking of changes within groups, leading to more robust and generalisable findings. Balanced datasets help draw reliable conclusions about longitudinal trends and patterns [56]. Nevertheless, we acknowledge that dropping participants can lead to missing information and selective bias. Therefore, our final supplementary analysis illustrates the AL trajectories using unbalanced data, allowing for a comparison with those derived from balanced data.

## Results

A total of 14,288 repeat observations, consisting of 53.70% females and 46.30% males, have been included in the baseline and the follow-up survey. *S2 Table* (see *Supporting Information*) summaries the demographic, socioeconomic, health status, and behavioural characteristics of older Chinese people for both sexes at baseline and 2015 survey.

*Table 1* collects the AL mean scores for each sex by different covariate groups. Females under 60 report lower AL mean scores than males, while females over 60 have higher scores than their male counterparts. It also highlights that participants with higher socio-economic status, i.e. those living in urban areas, wealthier and having higher educational backgrounds, report higher AL mean scores, indicating greater physiological dysregulation.

**Table 1:** Descriptive summaries of allostatic load mean scores (standard deviation) in different covariate groups by sex at baseline.

	Obs ^a^	All ^b^	Female ^b^	Male ^b^	
**Sex**	11792	1.199 (0.808)	1.163 (0.816)	1.244 (0.795)	
**Age**					
49 or younger	1408	1.061 (0.803)	0.980 (0.782)	1.228 (0.813)	
50–59	4145	1.193 (0.823)	1.134 (0.820)	1.271 (0.822)	
60–69	4206	1.236 (0.807)	1.214 (0.823)	1.259 (0.791)	
70–79	1743	1.233 (0.757)	1.292 (0.772)	1.182 (0.739)	
80 or older	290	1.240 (0.807)	1.334 (0.816)	1.164 (0.795)	
**Residence Type**					
Rural	9598	1.187 (0.802)	1.161 (0.814)	1.221 (0.785)	
Urban	1873	1.260 (0.822)	1.164 (0.828)	1.348 (0.805)	
**Marriage Status**					
Not married or separated	1910	1.202 (0.780)	1.184 (0.776)	1.235 (0.788)	
Married	9883	1.199 (0.811)	1.157 (0.821)	1.245 (0.796)	
**Wealth Tertials**					
Very or relatively rich	312	1.408 (0.798)	1.429 (0.825)	1.386 (0.772)	
Average	6134	1.203 (0.819)	1.175 (0.830)	1.238 (0.805)	
Poor or relatively poor	5182	1.182 (0.794)	1.133 (0.797)	1.237 (0.786)	
**Education**					
Elementary or lower	8121	1.187 (0.800)	1.180 (0.817)	1.199 (0.775)	
Middle school	2308	1.228 (0.818)	1.118 (0.820)	1.304 (0.808)	
High school	729	1.167 (0.839)	1.023 (0.780)	1.261 (0.863)	
College or higher	292	1.335 (0.825)	1.047 (0.780)	1.435 (0.817)	
**Smoking**					
Still have	3300	1.205 (0.785)	1.158 (0.810)	1.210 (0.782)	
Quit or never	8467	1.198 (0.818)	1.163 (0.817)	1.287 (0.812)	
**Drinking**					
More than once a month	2926	1.232 (0.795)	1.046 (0.770)	1.268 (0.795)	
Less than once a month	943	1.135 (0.767)	1.049 (0.737)	1.188 (0.780)	
Don’t drink	7915	1.195 (0.817)	1.180 (0.823)	1.232 (0.780)	
**Vigorous Exercise**				
Yes	2012	1.096 (0.784)	1.037 (0.802)	1.152 (0.814)	
No	3418	1.272 (0.812)	1.231 (0.808)	1.332 (0.814)	
**Num. Comorbidities**					
0	3466	1.044 (0.757)	0.970 (0.755)	1.124 (0.752)	
1	3392	1.204 (0.791)	1.173 (0.812)	1.241 (0.766)	
2	2296	1.226 (0.822)	1.188 (0.815)	1.273 (0.824)	
3 or more	2116	1.417 (0.838)	1.394 (0.839)	1.447 (0.834)	

Note: column

^a^ present the numbers of observations of participants, column

^b^ presents the mean score of AL to related rows.

To facilitate comprehension and comparison, we present the results of the random-intercept models and the supplementary analyses in *Table 2* (and *S3 Table*). *Model A* and *Model B* are our primary models, where AL is defined by clinical cut-off points. *Model B* includes an age-sex interaction term. Results from *Model C* and *Model D* are from the first supplementary analysis, where AL is defined by z-scores. *Model E* presents result for individuals born after the establishment of the People’s Republic of China (PRC), while *Model F* shows results for those born before the PRC. *Models G* and *H* display the results of our sensitivity analyses: *Model G* applies inverse probability weighting (IPW) instead of multiple imputations (MI) to the interaction model (*Model B*), and *Model H* uses z-scores to define AL and applies IPW (refer to *S3 Table in Supporting Information*). Please note that the results from *Models C* to *H* in *Tables 2* and *S3* are supplementary analyses to the primary models (*Models A* and *B*). They do not represent new findings.

**Table 2:** Maximum likelihood estimates for allostatic load: Coefficients and standard errors.

	Model A	Model B	Model C	Model D
**Age**	0.002 ± 0.001*†*	−0.004 ± 0.001 **	−0.003 ± 0.001 ***	−0.005 ± 0.001 ***
**Sex** (ref = male)	−0.099 ± 0.021***	−0.761 ± 0.120 ***	−0.070 ± 0.011 ***	−0.402 ± 0.056 ***
Age*sex		0.011 ± 0.002 ***		0.005 ± 0.001 ***
**Residence** (ref = urban)	−0.000 ± 0.020	0.002 ± 0.020	0.016 ± 0.010	0.018 ± 0.010 *†*
**Married** (ref = yes)	−0.001 ± 0.022	0.009 ± 0.022	−0.004 ± 0.010	0.000 ± 0.010
**Education** (ref = primary school)	0.017 ± 0.013	0.014 ± 0.013	0.013 ± 0.007 *†*	0.011 ± 0.007
**Wealth** (ref = wealthiest)	−0.053 ± 0.018 **	−0.054± 0.018 **	−0.023 ± 0.007 **	−0.021 ± 0.007 **
**Num. Comorbidities** (ref = 0)	0.112 ± 0.008 ***	0.111 ± 0.008 ***	0.056 ± 0.004 ***	0.055 ± 0.004 ***
**Current Smoker** (ref = yes)	−0.035 ± 0.02 *†*	−0.045 ± 0.021 *	−0.041 ± 0.009 ***	−0.044 ± 0.009 ***
**Drink more than once a month** (ref = yes)	0.035 ± 0.02 *†*	0.028 ± 0.021	0.043 ± 0.009 ***	0.038 ± 0.009 ***
**Vigorous Exercise** (ref = yes)	−0.145 ± 0.019 ***	−0.145 ± 0.019 ***	−0.044 ± 0.011**	−0.043 ± 0.011 **
*Constant*	1.172 ± 0.082 ***	1.534 ± 0.104 ***	0.180 ± 0.047	0.348 ± 0.054 ***

Model A: AL defined by clinical cut-points and top quartile with Multiple Imputation (MI): main random-intercept model.

Model B: AL defined by clinical cut-points and top quartile with MI: interaction random-intercept model.

Model C: AL defined by z-scores with MI: main random-intercept model.

Model D: AL defined by z-scores with MI: interaction random-intercept model.

ref, reference. sig:

†<0.1

*<0.05

**<0.01

***<0.001.

*Model A* in *Table 2* reveals that being older and male are associated with higher AL. The age-sex interaction term has been added to the interaction model (*Model B*) to assess differences in the age trajectories between males and females. Positive and statistically significant coefficients in the model indicate that age trajectories are significantly different between the sexes. The coefficients and standard errors presented in *Tables 2* and *S3* are the fixed part of the random intercept. For instance, the coefficient and standard error of sex is −0.761 ± 0.120 with males as the reference, meaning the expected (predicted) AL scores of females are 0.761 ± 0.120 points lower than that of males, controlling for other covariates (see *Model B*).

Our findings further show that wealthier Chinese people are more likely to have a higher AL. This finding contrasts with those in developed countries, such as the US and the UK, where wealthier people had a lower AL [3]. Being diagnosed with more comorbidities is associated with a higher AL. Smoking or engaging in more vigorous physical activities are negatively associated with AL. *Model C* and *Model D* in *Table 2* show similar results, suggesting robustness.

**Fig 1:**
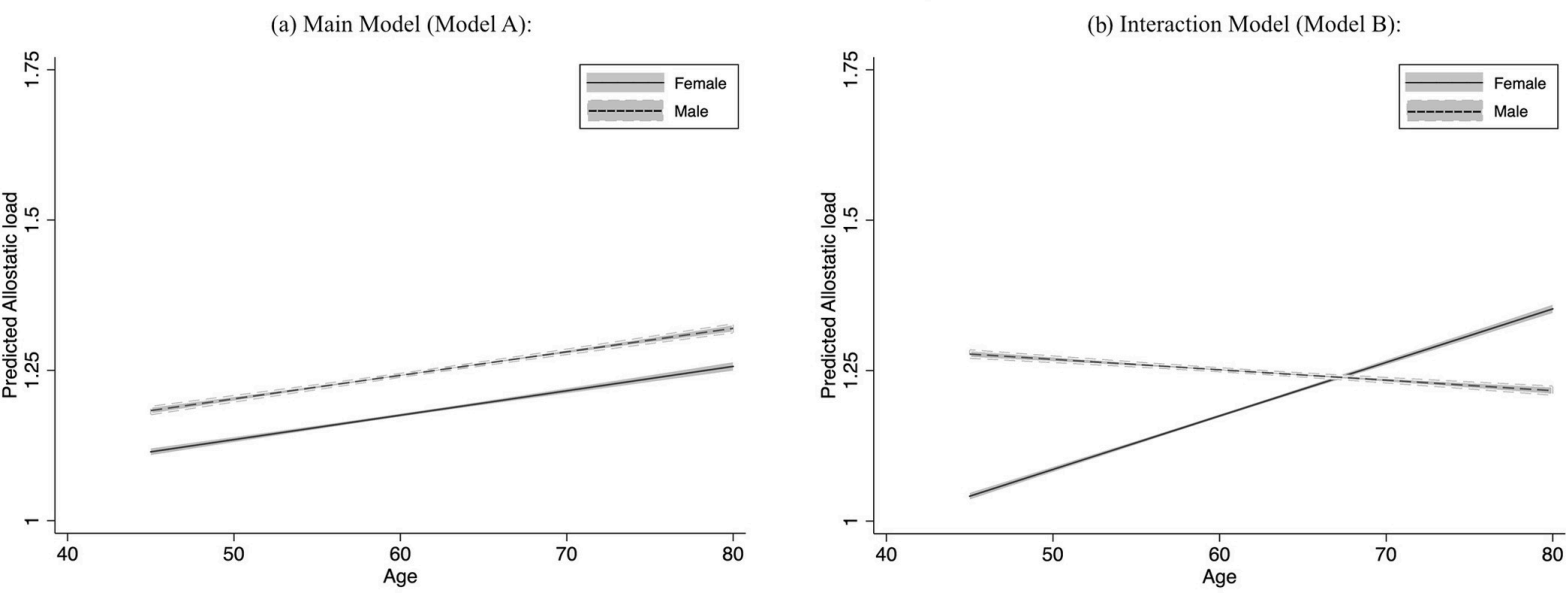
Predicted trajectories of allostatic load by sex: Main and interaction models. (a) illustrates that both females and males experience an increase in allostatic load (AL) as they age, with females maintaining consistently lower AL levels throughout their lives. (b) reveals that males have higher AL levels than females until around age 68. However, the gap between the sexes narrows over time, with females surpassing males in AL during their late 60s.

*Fig 1A and 1B* illustrates the age trajectories of AL for male and female adults in China. (*Fig 1A* shows the age trajectories for the main model (*Model A*), while *Fig 1B* displays those for the interaction model (*Model B*)). *Fig 1A* shows that females and males accumulate AL as they age, with females having a lower AL throughout their lives. *Fig 1B* shows that males have a higher AL than females until the males reach about 68 years of age. The differences in the levels of AL between females and males converge as they age, with females in their late 60s overtaking males.

**Fig 2:**
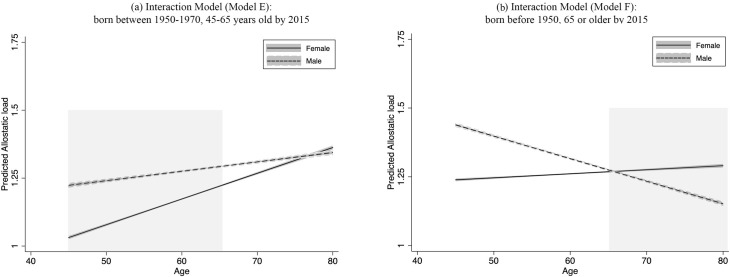
Predicted trajectories of allostatic load by sex: Age cohort before and after 1950: Interaction models. (a) illustrates AL increased with age for both males and females with females having an advantage in AL until around 76 years old for individuals who were born between 1950 and 1970. (b) shows trajectories for those born before 1950 characterised by a decline in AL for males and an increase for females.

We observe a slight declining trend in AL for males (see *Fig 1B*). To investigate this, we examine the AL trajectories for different age cohorts. For individuals born between 1950 and 1970, AL increased with age for both males and females (see *Fig 2A*), with females having an advantage in AL until around 76 years old. *Fig 2B* shows trajectories for those born before 1950 characterised by a decline in AL for males and an increase for females (Statistical results for *Fig 2A* and 2*B* are provided in *S3 Table*, *Models E* and *F in Supplementary Information*). Discussions on this age cohort difference will follow later.

## Discussion

Using nationally representative data, our study delineates the age trajectories of AL for midlife and older males and females in China. We find that the sex-specific trajectories converge with age, showing a female advantage until about the late 60s, after which the trajectory of females surpasses that of males (*Fig 1B*), suggesting a higher level of physiological dysregulation. Such a pattern is consistent with the phenomenon that females have a longer life expectancy but relatively poorer health status in later life.

One plausible explanation could be that estrogen has played a protective role in the female body in early life. Estrogen provides numerous benefits for females, including maintaining bone density, protecting cardiovascular health, regulating the menstrual cycle, and supporting reproductive function. It also positively impacts mood and mental well-being, and may help preserve cognitive function, potentially reducing the risk of osteoporosis, cardiovascular diseases, depression, anxiety, and neurodegenerative conditions [18]. The convergence in our study begins around age 45–50, likely linked to menopause. As reported, there is a gap of 5–10 years between the average age of menopause and noticeable accelerations of female physiological dysregulation [57]. As our trajectories show, females maintain an advantage in AL for years until their late 60s. Previous research also reflects such latency of physiological disorders in the absence of estrogen [58]. During menopause, the ovaries gradually produce less estrogen, leading to a range of physical and emotional symptoms. Long-term effects include an increased risk of osteoporosis due to decreased bone density, a higher risk of cardiovascular disease, and potential cognitive changes. The reduction in estrogen can also affect mood, leading to symptoms such as depression and anxiety. Eventually, the level of AL in females surpasses that of males with declining estrogen levels, leading to a worse health status in later life.

Further, the widely observed female advantage in lifespan and in AL may have a fundamental genetic root beyond the human species. Research has revealed that the heterogametic sex (e.g. male humans) tends to have a significantly shorter lifespan (17.6%), compared to the homogametic sex (e.g. female humans). The absent chromosome (only x) in the heterogametic sex exposes the males to deleterious mutations on the sex chromosome [59]. To sum up, the reasons for females’ advantage in life expectancy may be multifactorial but any revealed factor, in this case AL, will help us to better understand the mechanisms of ageing and longevity from the perspective of physiological dysregulation.

We highlight an unusually slight declining trajectory of AL for males later in life (see *Fig 1B*). AL, as a marker of accumulated biological risk and physiological dysregulation, is expected to increase with age. Studies have reported that AL and various biomarkers related to AL increased with age among older people in England [60] and Taiwan [61]. The decline in AL among males with age suggests a rejuvenation in later life, which seems less plausible.

Survival bias could contribute to this unusual decline because individuals who survive into older age are often healthier than those who do not, skewing the data. In other words, the less healthy individuals with higher AL may not live as long, leaving a population of older males who are comparatively healthier and thus, exhibit lower AL. In our study, males older than 70 report higher AL mean scores than their female counterparts (see *Table 1*), a reversed pattern in younger groups. These selected survivors can create the appearance of a decline in AL among the oldest males, despite the general trend of increasing AL with age.

Early-life nutrition and stress could also lead to different health outcomes in later life [50]. Consequently, we investigate the potential presence of age cohorts among midlife and older adults in China. We find that for Chinese people born in 1950 and later, AL increases with age for both sexes, but females exhibit lower AL levels overall (see *Fig 2A*). The two trajectories are projected to converge when the people are about 75 years old, which is the average life expectancy of Chinese people [35]. Such changes in AL with age are consistent with those in the UK [3], and the pattern of life expectancy worldwide.

In contrast, we observe a gentle increase in AL with age for females and more importantly, a drastic decline in AL among older males (see *Fig 2B*). This decrease in AL with age among males elucidates where the overall decline originates. Generations of turmoil, wars and an extreme shortage of healthcare before the establishment of the republic in 1949 led to a generation of males with an estimated average life expectancy of 44.6 years by 1950 [62]. By 2015, the majority in this pre-establishment cohort had died and left a small but highly selected healthier cohort.

Our results show a positive association between wealth and AL. This finding contradicts those observed in developed countries. People living in an advantageous socioeconomic status (SES) have health advantages in developed countries, such as the US and the UK [3]. In contrast, people in advantageous SES in emerging economies tend to engage in unhealthy behaviours, being physically inactive and obese [63, 64]. Further, the wealthy tend to engage in sedentary work that requires less energy, and their health worsens by having more access to surplus nutrition [65].

The major limitation of the present study is that we cannot completely account for the decline in the AL of Chinese adult males. A recently published study on urban-rural disparities in physiological health also identified a similar, sightly declining age trajectory of AL for males, utilising data from CHARLS. Regrettably, the study did not provide further discussion on this topic [38]. In addition to survival bias, our study suggests that there is an age cohort of Chinese males who were born before 1950 that may be responsible for the declining trend. A combination of MI and IPW [66] or joint models for attritions [67] may be solutions to such declining pattern, but they are impractical to use due to the limitations of the current datasets (being only two surveys for inclusion in the joint models). Furthermore, the cohort comparison needs to be very carefully interpreted in terms of whether the trajectories of those born before 1950 are different from those born later. The present modelling is based on different age cohorts, and the older cohort are more likely to drop out. Hence, the estimates may not necessarily be comparable to the younger age cohort. To make it a strict birth cohort study, the two age cohorts need to be studied at the same age. Nevertheless, this will only be possible when the participants have been followed up for 20 years for instance. To estimate change over time the study would also require a third wave so that the later cohort could have a repeated measure. Although it is possible to estimate the linear growth covering earlier and later years than the actual age of the two cohorts, readers should bear in mind that the model is mixing cohort and age effects.

In addition, direct measures of the stress-response system, such as urinary norepinephrine, epinephrine and salivary cortisol, are not available. Other biomarkers like fibrinogen, peak expiratory flow, and hip measurements, were not collected by CHARLS. Nevertheless, our methods satisfy the requirement that "information on at least four of five subsystems had to be available to calculate the score" [41]. Likewise, some covariates that are potentially related to AL are not included, such as family background or the work stress people experience in early adulthood [34]. Thus, a life history study is desirable. Also, due to data limitation of some instruments used in the study, the results should be interpreted carefully. For instance, self-reports on drinking frequency without a measure of the quantity (units) can be affected by social desirability or subjective self-rated wealth, which is subject to participants’ own life experience (see *S3 Table*).

The present study has some strengths. To our knowledge, it is the first empirical study to depict the sex-specific age trajectories of AL among midlife and older Chinese adults, based on a large and nationally representative dataset. The study highlights converging AL trajectories with a female advantage [3], rather than a male advantage [15].

Secondly, despite its limitations, we conducted a series of supplementary analyses to ensure the robustness of our findings. When applying IPW to the model, the results (see *Model G* in *S3 Table*) are comparable to those obtained from the multiply imputed model (*Model B*). The patterns of the AL trajectories (see *S1A Fig*), delineated based on *Model G*, are consistent with those in *Fig 1B*: a rising AL trajectory for females and a slightly declining trajectory for males. Furthermore, *S2 and S3 Figs (see Supporting Information)* reaffirm the presence of a healthier cohort. Through all these analyses, we confirm that there is a declining trajectory of AL for males based on data from CHARLS, irrespective of the underlying causes. This finding suggests that further research on AL and other health-related studies needs to account for the potentially healthier cohort, especially when using CHARLS. More waves of biomarker sample collection are desired to address these limitations.

## Conclusions

This study delineates the trajectories of AL among midlife and older adults in China, one of the largest developing countries and a non-Western context. It also seeks to interpret the contradiction between the female advantage in life expectancy and their disadvantage in health in later life from the perspective of physiological dysregulation. We find that the sex-specific trajectories converge with age, with females surpassing males around their late 60s. This pattern aligns with the observed female advantage in life expectancy but highlights their loss of health advantages in later life. Our findings underscore the presence of a healthier older male cohort in CHARLS, suggesting that future studies should consider this factor in their analyses.

## Supporting information

S1 Table Specification of survey instruments. (PDF)

S2 Table Descriptive summaries of samples from 2011 survey (baseline) and 2015 survey. (PDF)

S3 Table Maximum likelihood estimates for allostatic load: Coefficients and standard errors. (PDF)

S1 Fig Predicted trajectories of allostatic load by gender: Interaction models. (PDF)

S2 Fig Predicted trajectories of allostatic load by gender: Socio-historical cohort before and after 1950: Interaction models. (PDF)

S3 Fig Predicted trajectories of allostatic load by gender: Unbalanced data. (PDF)

S1 Checklist STROSA (version 2) checklist. (PDF)

## References

[1] Mathers CD, Loncar D. Projections of global mortality and burden of disease from 2002 to 2030. PLoS Med. 2006;3(11):e442. doi: 10.1371/journal.pmed.0030442 17132052 PMC1664601

[2] Pardue ML, Wizemann TM. Exploring the biological contributions to human health: does sex matter? National Academies Press; 2001. 25057540

[3] Tampubolon G, Maharani A. Trajectories of allostatic load among older Americans and Britons: longitudinal cohort studies. BMC Geriatr. 2018;18(1):1–10. 30352552 10.1186/s12877-018-0947-4 PMC6199736

[4] Rieker PP, Bird CE. Rethinking gender differences in health: why we need to integrate social and biological perspectives. Journals Gerontol Ser B Psychol Sci Soc Sci. 2005;60(Special Issue 2):S40—S47. doi: 10.1093/geronb/60.special_issue_2.s40 16251589

[5] Leveille SG, Penninx BW, Melzer D, Izmirlian G, Guralnik JM. Sex differences in the prevalence of mobility disability in old age: the dynamics of incidence, recovery, and mortality. Journals Gerontol Ser B. 2000;55(1):S41—S50. doi: 10.1093/geronb/55.1.s41 10728129

[6] Murtagh KN, Hubert HB. Gender differences in physical disability among an elderly cohort. Am J Public Health. 2004;94(8):1406–11. doi: 10.2105/ajph.94.8.1406 15284051 PMC1448463

[7] Colledge NR, Cantley P, Peaston I, Brash H, Lewis S, Wilson JA. Ageing and balance: the measurement of spontaneous sway by posturography. Gerontology. 1994;40(5):273–8. doi: 10.1159/000213596 7959084

[8] Hageman PA, Leibowitz JM, Blanke D. Age and gender effects on postural control measures. Arch Phys Med Rehabil. 1995;76(10):961–5. doi: 10.1016/s0003-9993(95)80075-1 7487439

[9] Musselman K, Brouwer B. Gender-related differences in physical performance among seniors. J Aging Phys Act. 2005;13(3):239–53. doi: 10.1123/japa.13.3.239 16192652

[10] Prudham D, Evans JG. Factors associated with falls in the elderly: a community study. Age Ageing. 1981;10(3):141–6. doi: 10.1093/ageing/10.3.141 7270321

[11] Wolfson L, Whipple R, Derby CA, Amerman P, Nashner L. Gender differences in the balance of healthy elderly as demonstrated by dynamic posturography. J Gerontol. 1994;49(4):M160—M167. doi: 10.1093/geronj/49.4.m160 8014390

[12] Caspersen CJ, Pereira MA, Curran KM. Changes in physical activity patterns in the United States, by sex and cross-sectional age. Med \& Sci Sport \& Exerc. 2000;32(9):1601–9. doi: 10.1097/00005768-200009000-00013 10994912

[13] Heyward VH, Wagner DR. Applied Body Composition Assessment. Human Kinetics; 2004.

[14] Vogel JA, Patton JF, Mello RP, Daniels WL. An analysis of aerobic capacity in a large United States population. J Appl Physiol. 1986;60(2):494–500. doi: 10.1152/jappl.1986.60.2.494 3949654

[15] Yang Y, Kozloski M. Sex differences in age trajectories of physiological dysregulation: inflammation, metabolic syndrome, and allostatic load. Journals Gerontol Ser A Biomed Sci Med Sci. 2011;66(5):493–500. doi: 10.1093/gerona/glr003 21350248 PMC3107023

[16] McEwen BS. Sex, stress and the hippocampus: allostasis, allostatic load and the aging process. Neurobiol Aging. 2002;23(5):921–39. doi: 10.1016/s0197-4580(02)00027-1 12392796

[17] Galea LAM. Exposure to predator odor suppresses cell proliferation in the dentate gyrus of adult rats via cholinergic mechanism. In: Soc Neurosci. 1996. p. 1196.

[18] Galea LAM, McEwen BS, Tanapat P, Deak T, Spencer RL, Dhabhar FS. Sex differences in dendritic atrophy of CA3 pyramidal neurons in response to chronic restraint stress. Neuroscience. 1997;81(3):689–97. doi: 10.1016/s0306-4522(97)00233-9 9316021

[19] Seeman TE, McEwen BS, Singer BH, Albert MS, Rowe JW. Increase in urinary cortisol excretion and memory declines: MacArthur studies of successful aging. J Clin Endocrinol Metab. 1997;82(8):2458–65. doi: 10.1210/jcem.82.8.4173 9253318

[20] Fried LP, Ferrucci L, Darer J, Williamson JD, Anderson G. Untangling the concepts of disability, frailty, and comorbidity: implications for improved targeting and care. Journals Gerontol Ser A Biol Sci Med Sci. 2004;59(3):M255–63. doi: 10.1093/gerona/59.3.m255 15031310

[21] Crimmins EM, Seeman TE. Integrating biology into the study of health disparities. Popul Dev Rev. 2004;30:89–107.

[22] Crimmins EM, Johnston M, Hayward M, Seeman T. Age differences in allostatic load: an index of physiological dysregulation. Exp Gerontol. 2003;38(7):731–4. doi: 10.1016/s0531-5565(03)00099-8 12855278

[23] Seeman TE, McEwen BS, Rowe JW, Singer BH. Allostatic load as a marker of cumulative biological risk: MacArthur studies of successful aging. Proc Natl Acad Sci. 2001;98(8):4770–5. doi: 10.1073/pnas.081072698 11287659 PMC31909

[24] Malik S, Wong ND, Franklin SS, Kamath T V, L’Italien GJ, Pio JR, et al. Impact of the metabolic syndrome on mortality from coronary heart disease, cardiovascular disease, and all causes in United States adults. Circulation. 2004;110(10):1245–50. doi: 10.1161/01.CIR.0000140677.20606.0E 15326067

[25] Khera A, McGuire DK, Murphy SA, Stanek HG, Das SR, Vongpatanasin W, et al. Race and gender differences in C-reactive protein levels. J Am Coll Cardiol. 2005;46(3):464–9. doi: 10.1016/j.jacc.2005.04.051 16053959

[26] Kim JK, Alley D, Seeman T, Karlamangla A, Crimmins E. Recent changes in cardiovascular risk factors among women and men. J Women’s Heal. 2006;15(6):734–46. doi: 10.1089/jwh.2006.15.734 16910905

[27] Lakoski SG, Cushman M, Criqui M, Rundek T, Blumenthal RS, D’Agostino RB Jr, et al. Gender and C-reactive protein: data from the Multiethnic Study of Atherosclerosis (MESA) cohort. Am Heart J. 2006;152(3):593–8. doi: 10.1016/j.ahj.2006.02.015 16923436

[28] Loucks EB, Berkman LF, Gruenewald TL, Seeman TE. Relation of social integration to inflammatory marker concentrations in men and women 70 to 79 years. Am J Cardiol. 2006;97(7):1010–6. doi: 10.1016/j.amjcard.2005.10.043 16563907

[29] Halvorsen DS, Johnsen SH, Mathiesen EB, Njølstad I. The association between inflammatory markers and carotid atherosclerosis is sex dependent: the Tromsø Study. Cerebrovasc Dis. 2009;27(4):392–7. 19276622 10.1159/000207443

[30] Goldman N, Weinstein M, Cornman J, Singer B, Seeman T, Goldman N, et al. Sex differentials in biological risk factors for chronic disease: Estimates from population-based surveys. J women’s Heal. 2004;13(4):393–403. doi: 10.1089/154099904323087088 15186656

[31] Seeman TE, Crimmins E, Huang MH, Singer B, Bucur A, Gruenewald T, et al. Cumulative biological risk and socio-economic differences in mortality: MacArthur studies of successful aging. Soc Sci Med. 2004;58(10):1985–97. doi: 10.1016/S0277-9536(03)00402-7 15020014

[32] Nakamura E, Miyao K. Sex differences in human biological aging. Journals Gerontol Ser A Biol Sci Med Sci. 2008;63(9):936–44. doi: 10.1093/gerona/63.9.936 18840798

[33] Honkalampi K, Virtanen M, Hintsa T, Ruusunen A, Mäntyselkä P, Ali-Sisto T, et al. Comparison of the level of allostatic load between patients with major depression and the general population. J Psychosom Res. 2021;143:110389. doi: 10.1016/j.jpsychores.2021.110389 33609985

[34] Sun J, Wang S, Zhang JQ, Li W. Assessing the cumulative effects of stress: The association between job stress and allostatic load in a large sample of Chinese employees. Work \& Stress. 2007;21(4):333–47.

[35] World Health Organization. China country assessment report on ageing and health. World Health Organization; 2015.

[36] Mao F, Astell-Burt T, Feng X, Liu Y, Dong J, Liu S, et al. Social and spatial inequalities in allostatic load among adults in China: a multilevel longitudinal study. BMJ Open. 2019;9(11):e031366. doi: 10.1136/bmjopen-2019-031366 31784439 PMC6924714

[37] Xu H. Multilevel socioeconomic differentials in allostatic load among Chinese adults. Health Place. 2018;53:182–92. doi: 10.1016/j.healthplace.2018.08.012 30172822 PMC6150819

[38] Ye X, Zhu D, Ding R, He P. Association of life‐course socioeconomic status with allostatic load in Chinese middle‐aged and older adults. Geriatr Gerontol Int. 2022;22(5):425–32. doi: 10.1111/ggi.14373 35285137

[39] Zhao Y, Hu Y, Smith JP, Strauss J, Yang G. Cohort profile: the China health and retirement longitudinal study (CHARLS). Int J Epidemiol. 2014;43(1):61–8. doi: 10.1093/ije/dys203 23243115 PMC3937970

[40] Chen X, Crimmins E, Hu P, Kim JK, Meng Q, Strauss J, et al. Venous blood-based biomarkers in the China health and retirement longitudinal study: rationale, design, and results from the 2015 wave. Am J Epidemiol. 2019;188(11):1871–7. doi: 10.1093/aje/kwz170 31364691 PMC6825825

[41] Read S, Grundy E. Allostatic load and health in the older population of England: a crossed-lagged analysis. Psychosom Med. 2014;76(7):490. doi: 10.1097/PSY.0000000000000083 25153937 PMC4418773

[42] Karlamangla AS, Singer BH, McEwen BS, Rowe JW, Seeman TE. Allostatic load as a predictor of functional decline: MacArthur studies of successful aging. J Clin Epidemiol. 2002;55(7):696–710. 12160918 10.1016/s0895-4356(02)00399-2

[43] Gruenewald TL, Karlamangla AS, Hu P, Stein-Merkin S, Crandall C, Koretz B, et al. History of socioeconomic disadvantage and allostatic load in later life. Soc Sci \& Med. 2012;74(1):75–83. doi: 10.1016/j.socscimed.2011.09.037 22115943 PMC3264490

[44] Prasad K. C-reactive protein (CRP)-lowering agents. Cardiovasc Drug Rev. 2006;24(1):33–50. doi: 10.1111/j.1527-3466.2006.00033.x 16939632

[45] Stephan Y, Sutin AR, Luchetti M, Terracciano A. Allostatic load and personality: A 4-year longitudinal study. Psychosom Med. 2016;78(3):302. doi: 10.1097/PSY.0000000000000281 26716813 PMC5481782

[46] Kaneda T, Zimmer Z, Tang Z. Differentials in life expectancy and active life expectancy by socioeconomic status among older adults in Beijing. 2004. 10.1080/09638280400006481 16025751

[47] Kaneda T, Zimmer Z, Tang Z. Socioeconomic status differentials in life and active life expectancy among older adults in Beijing. Disabil Rehabil. 2004. 10.1080/09638280400006481 16025751

[48] Rabe-Hesketh S, Skrondal A. Multilevel and longitudinal modeling using Stata. STATA press; 2008.

[49] Azur MJ, Stuart EA, Frangakis C, Leaf PJ. Multiple imputation by chained equations: what is it and how does it work? Int J Methods Psychiatr Res. 2011;20(1):40–9. doi: 10.1002/mpr.329 21499542 PMC3074241

[50] Tampubolon G. Growing up in poverty, growing old in infirmity: the long arm of childhood conditions in Great Britain. PLoS One. 2015;10(12):e0144722. doi: 10.1371/journal.pone.0144722 26675009 PMC4682716

[51] Gao M, Kuang W, Qiu P, Wang H, Lv X, Yang M. The time trends of cognitive impairment incidence among older Chinese people in the community: based on the CLHLS cohorts from 1998 to 2014. Age Ageing. 2017;46(5):787–93. doi: 10.1093/ageing/afx038 28369164

[52] Cole SR, Hernán MA. Constructing inverse probability weights for marginal structural models. Am J Epidemiol. 2008;168(6):656–64. doi: 10.1093/aje/kwn164 18682488 PMC2732954

[53] Gottesman RF, Rawlings AM, Sharrett AR, Albert M, Alonso A, Bandeen-Roche K, et al. Impact of differential attrition on the association of education with cognitive change over 20 years of follow-up: the ARIC neurocognitive study. Am J Epidemiol. 2014;179(8):956–66. doi: 10.1093/aje/kwu020 24627572 PMC3966720

[54] Weuve J, Tchetgen EJT, Glymour MM, Beck TL, Aggarwal NT, Wilson RS, et al. Accounting for bias due to selective attrition: the example of smoking and cognitive decline. Epidemiology. 2012;23(1):119. doi: 10.1097/EDE.0b013e318230e861 21989136 PMC3237815

[55] Hoffman L. Longitudinal analysis: Modeling within-person fluctuation and change. Routledge; 2015.

[56] Twisk JWR. Applied longitudinal data analysis for epidemiology: a practical guide. cambridge university press; 2013.

[57] Horiuchi S. Postmenopausal acceleration of age-related mortality increase. Journals Gerontol Ser A Biol Sci Med Sci. 1997;52(1):B78–92. doi: 10.1093/gerona/52a.1.b78 9008661

[58] Santen RJ, Allred DC, Ardoin SP, Archer DF, Boyd N, Braunstein GD, et al. Postmenopausal hormone therapy: an Endocrine Society scientific statement. J Clin Endocrinol Metab. 2010;95(7_supplement_1):s1–66. doi: 10.1210/jc.2009-2509 20566620 PMC6287288

[59] Xirocostas ZA, Everingham SE, Moles AT. The sex with the reduced sex chromosome dies earlier: a comparison across the tree of life. Biol Lett. 2020;16(3):20190867. doi: 10.1098/rsbl.2019.0867 32126186 PMC7115182

[60] van Deurzen I, Vanhoutte B. A longitudinal study of allostatic load in later life: The role of sex, birth cohorts, and risk accumulation. Res Aging. 2019;41(5):419–42. doi: 10.1177/0164027518813839 30466351

[61] Glei DA, Goldman N, Rodríguez G, Weinstein M. Beyond self-reports: changes in biomarkers as predictors of mortality. Popul Dev Rev. 2014;40(2):331–60. doi: 10.1111/j.1728-4457.2014.00676.x 25089065 PMC4117355

[62] United Nation. World population ageing 2013. Department of Economic and Social Affairs PD; 2013.

[63] Hanandita W, Tampubolon G. The double burden of malnutrition in Indonesia: Social determinants and geographical variations. SSM-population Heal. 2015;1:16–25. doi: 10.1016/j.ssmph.2015.10.002 29349117 PMC5757754

[64] Roemling C, Qaim M. Obesity trends and determinants in Indonesia. Appetite. 2012;58(3):1005–13. doi: 10.1016/j.appet.2012.02.053 22402303

[65] World Health Organization. Diet, nutrition, and the prevention of chronic diseases: report of a joint WHO/FAO expert consultation. Vol. 916. World Health Organization; 2003. 12768890

[66] Seaman SR, White IR, Copas AJ, Li L. Combining multiple imputation and inverse‐probability weighting. Biometrics. 2012;68(1):129–37. doi: 10.1111/j.1541-0420.2011.01666.x 22050039 PMC3412287

[67] Crowther MJ, Abrams KR, Lambert PC. Joint modeling of longitudinal and survival data. Stata J. 2013;13(1):165–84.

