# Supplementary material for: Sex differences in allostatic load trajectories among midlife and older adults: Evidence from the China health and retirement longitudinal study
Source: PLoS One. 2024 Dec 26;19(12):e0315594. doi: 10.1371/journal.pone.0315594 (PMC11670931; doi:10.1371/journal.pone.0315594)
Supplement: S1 Table — (PDF) [file pone.0315594.s001.pdf]

S1 Table: Specification of survey instruments

Instruments	Specification
**Residence Type**	Urban/rural indicator is based on participants' Hukou (an official residence registration system). Data are automatically obtained based on the location the survey taken place.
**Marital Status**	Self-reported marital status.
**Wealth Tertiles**	Due to massive non-responses of objective measurements of individual wealth (e.g. annual income) in CHARLS, we decided to use subjective measurement of individual wealth and quality of life, which has little missing values. Interviewees were asked to rate in their own opinions if the life standards described in two vignettes are: very high, high, average, relatively poor or poor, respectively. Then they were asked to rate their own wealth and life quality.
**Education Background**	10-level self-reported school experience. They are: 1. No formal education / illiterate. 2. Did not finish primary school but capable of reading or writing. 3. Sishu (traditional Chinese school). 4. Elementary school. 5. Middle school. 6. High school. 7. Vocational school. 8. Two/Three Year College / associate degree. 9. Four Year College / bachelor's degree. 10. post-graduate / master's degree or above.
**Num. Comorbidities**	14 comorbidities were included in the study based on doctor's diagnosis. They are:<br>1. Hypertension, 2. Dyslipidemia, 3. Diabetes or High Blood Sugar, 4. Cancer or Malignant Tumour, 5. Chronic Lung Diseases, 6. Liver Disease, 7. Heart Problems, 8. Stroke, 9. kidney disease, 10. Stomach or Other Digestive Disease, 11. Emotional, Nervous, or Psychiatric Problems, 12. Memory-Related Disease, 13. Arthritis or Rheumatism and 14. Asthma.
**Smoking Behaviours**	Participants were asked if they: 1. Still smoke, 2. Quit or Never smoke.
**Drinking Behaviours**	Drinking is measured based on frequency instead of quantity in this study.
**Physical activities**	Self-reported physical activities. Participants were asked if they have been engaging in activities that makes them breathe much harder than normal and may include heavy lifting, digging, plowing, aerobics, fast bicycling, and cycling with a heavy load at least 10 minutes at a time in a usual week.
