# Supplementary material for: Sex differences in allostatic load trajectories among midlife and older adults: Evidence from the China health and retirement longitudinal study
Source: PLoS One. 2024 Dec 26;19(12):e0315594. doi: 10.1371/journal.pone.0315594 (PMC11670931; doi:10.1371/journal.pone.0315594)
Supplement: S2 Table — (PDF) [file pone.0315594.s002.pdf]

S2 Table: Descriptive summaries of samples from 2011 survey (baseline) and 2015 survey

	Baseline Survey (Wave 1)				2015 Survey (Wave 4)			
	**N (%)**	**Female (%)**	**Male (%)**	**.p**	**N (%)**	**Female (%)**	**Male (%)**	.p
**Gender**	7144	3836 (53.70)	3308 (46.30)		7144	3836 (53.70)	3308 (46.30)	
**Age Group**	7143	3835	3308	0.000	7143	3835	3308	0.000
49 or younger	1327 (18.85)	820 (21.38)	507 (15.33)		177 (2.48)	109 (2.84)	68 (2.06)	
50-59	2638 (36.93)	1444 (37.65)	1194 (36.04)		2506 (35.08)	1459 (38.04)	1047 (31.65)	
60-69	2256 (31.58)	1133 (29.54)	1123 (33.95)		2877 (40.28)	1505 (39.24)	1372 (41.48)	
70-79	820 (11.48)	384 (10.01)	436 (13.18)		1293 (18.10)	621 (16.19)	672 (20.31)	
80 or older	102 (1.43)	54 (1.41)	48 (1.45)		290 (4.06)	141 (3.68)	149 (4.50)	
**Residence Type**	7138	3833	3305	0.000	6845	3677	3168	0.000
Agricultural (Rural)	5974 (83.69)	3280 (85.57)	2694 (81.85)		5431 (79.34)	2986 (81.21)	2445 (77.18)	
Non-agricultural (Urban)	1130 (15.83)	535 (13.96)	595 (18.00)		1356 (19.81)	658 (17.90)	698 (22.03)	
Others (Unified)	34 (0.48)	18 (0.47)	16 (0.48)		58 (0.58)	33 (0.90)	25 (0.79)	
**Marital Status**	7140	3833	3307	0.000	7143	3836	3307	0.000
not married or separated	1088 (15.24)	693 (18.08)	395 (11.94)		1303 (18.24)	857 (22.34)	446 (13.49)	
married	6052 (84.76)	3140 (81.92)	2912 (88.06)		5840 (81.76)	2979 (77.66)	2861 (86.51)	
**Wealth Tertiles**	6871	3704	3167	0.101	6871	3704	3167	0.101
Very or relatively rich	185 (2.69)	91 (2.46)	94 (2.97)		185 (2.69)	91 (2.46)	94 (2.97)	
Average	3628 (52.80)	1995 (53.86)	1633 (51.56)		3628 (52.80)	1995 (53.86)	1633 (51.56)	
Poor or relatively poor	3058 (44.51)	1618 (43.68)	1440 (45.47)		3058 (44.51)	1618 (43.68)	1440 (45.47)	
**Education Background**	7135	3830	3305	0.000	6719	3625	3094	0.000
Elementary or lower	5002 (70.11)	3009 (78.56)	1993 (60.30)		4708 (70.07)	2844 (78.46)	1864 (60.25)	
Middle school	1452 (20.35)	573 (14.96)	879 (26.60)		1364 (20.30)	539 (14.87)	825 (26.66)	
High school	469 (6.57)	190 (4.96)	279 (8.44)		449 (6.68)	185 (5.10)	264 (8.53)	
College or higher	212 (2.97)	58 (1.51)	154 (4.66)		198 (2.95)	57 (1.57)	141 (4.56)	
**Num. Comorbidities**	6798	3645	3153	0.004	6798	3645	3153	0.004
0	2137 (31.44)	1092 (29.96)	1045 (33.14)		2137 (31.44)	1092 (29.96)	1045 (33.14)	
1	2029 (29.85)	1079 (29.60)	950 (30.13)		2029 (29.85)	1079 (29.60)	950 (30.13)	
2	1354 (19.92)	741 (20.33)	613 (19.44)		1354 (19.92)	741 (20.33)	613 (19.44)	
3 or more	1278 (18.80)	733 (20.11)	545 (17.29)		1278 (18.80)	733 (20.11)	545 (17.29)	
**Smoking Behaviours**	7023	3814	3209	0.000	7139	3836	3303	0.000
Still have	2112 (30.07)	225 (5.90)	1533 (46.50)		1928 (27.01)	205 (5.34)	1723 (52.16)	
Quit or never	4911 (69.93)	3589 (94.10)	1322 (41.20)		5211 (72.99)	3631 (94.66)	1580 (47.84)	
**Drinking Behaviours**	7120	3823	3297	0.000	7139	3834	3305	0.000
More than Once a Month	1801 (25.29)	268 (7.01)	1533 (46.50)		1813 (25.40)	301 (7.85)	1512 (45.75)	
Less than Once a Month	570 (8.01)	186 (4.87)	384 (11.65)		597 (8.36)	247 (6.44)	350 (10.59)	
Don't drink	4749 (66.70)	3369 (88.12)	1380 (41.86)		4729 (66.24)	3286 (85.71)	1443 (43.66)	
**Physical activities**	2979	1665	1314	0.000	3518	1900	1618	0.000
No	1892 (63.51)	1159 (69.61)	733 (55.78)		2268 (64.47)	1325 (69.74)	943 (58.28)	
Yes	1087 (36.49)	506 (30.39)	581 (44.22)		1250 (35.53)	575 (30.26)	675 (41.72)	
