# Supplementary material for: Sex differences in allostatic load trajectories among midlife and older adults: Evidence from the China health and retirement longitudinal study
Source: PLoS One. 2024 Dec 26;19(12):e0315594. doi: 10.1371/journal.pone.0315594 (PMC11670931; doi:10.1371/journal.pone.0315594)
Supplement: S3 Table — (PDF) [file pone.0315594.s003.pdf]

S3 Table: Maximum likelihood estimates for allostatic load: coefficients and standard errors

	Model E	Model F	Model G	Model H
**Age**	0.001± 0.003	−0.010 ± 0.003 **	−0.005 ± 0.004	−0.008 ± 0.001***
**Sex** (ref = male)	−0.536 ± 0.214 *	−0.647 ± 0.304*	−0.768 ± 0.294 **	−0.636 ± 0.081***
**Age*gender**	0.007 ± 0.004 †	0.009 ± 0.004*	0.011 ± 0.005 *	0.009 ± 0.001***
**Residence** (ref = urban)	−0.014 ± 0.027	0.026 ± 0.032	0.031 ± 0.052	0.018 ± 0.015
**Married** (ref = yes)	0.064 ± 0.030 *	−0.070 ± 0.016 *	0.021 ± 0.053	0.027 ± 0.015†
**Education** (ref = primary school)	0.015 ± 0.017	0.027 ± 0.022	0.022 ± 0.021	0.008 ± 0.009
**Wealth** (ref = wealthiest)	−0.076 ± 0.023**	−0.015± 0.026	−0.062 ± 0.023 **	−0.014 ± 0.010
**Num. Comorbidities** (ref = 0)	0.122 ± 0.010 ***	0.091 ± 0.012 ***	0.121 ± 0.012 ***	0.054 ± 0.005 ***
**Current Smoker** (ref = yes)	−0.050 ± 0.027 †	−0.047 ± 0.031	−0.055 ± 0.046	−0.044 ± 0.014 **
**Drink more than once a month** (ref = yes)	0.025 ± 0.024	0.029 ± 0.033	0.053 ± 0.050	0.049 ± 0.013***
**Vigorous Exercise** (ref = yes)	−0.137 ± 0.025 ***	−0.167 ± 0.026 ***	−0.095 ± 0.035 **	−0.056 ± 0.011***
Constant	1.588 ± 0.496 ***	1.966 ± 0.238 ***	1.588 ± 0.496 ***	0.498 ± 0.075***

Model E: AL defined by clinical cut-points and top quartile with MI (age cohort: 45-65): interaction random-intercept model.

Model F: AL defined by clinical cut-points and top quartile with MI (age cohort: 65 or older): interaction random-intercept model.

Model G: AL defined by clinical cut-points and top quartile with IPW: interaction random-intercept model.

Model H: AL defined by z-scores with IPW: interaction random-intercept model.

ref, reference. sig: †<0.1; *<0.05; **<0.01; ***<0.001.
