# Supplementary material for: Sex differences in allostatic load trajectories among midlife and older adults: Evidence from the China health and retirement longitudinal study
Source: PLoS One. 2024 Dec 26;19(12):e0315594. doi: 10.1371/journal.pone.0315594 (PMC11670931; doi:10.1371/journal.pone.0315594)

## S1 Fig: Predicted trajectories of allostatic load by gender: interaction models

(a) Weighted Interaction Model (Model G)

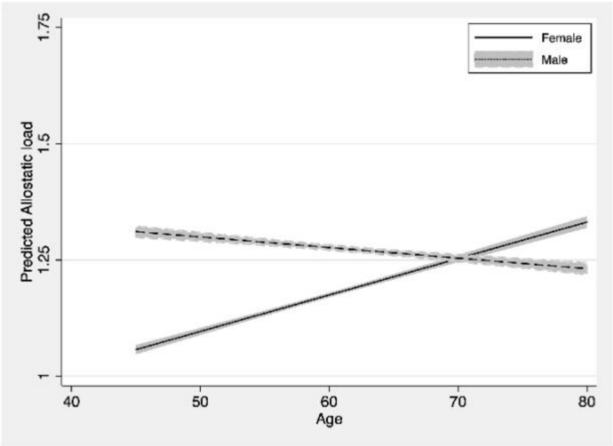


(b) Weighted Interaction Model (Model H)

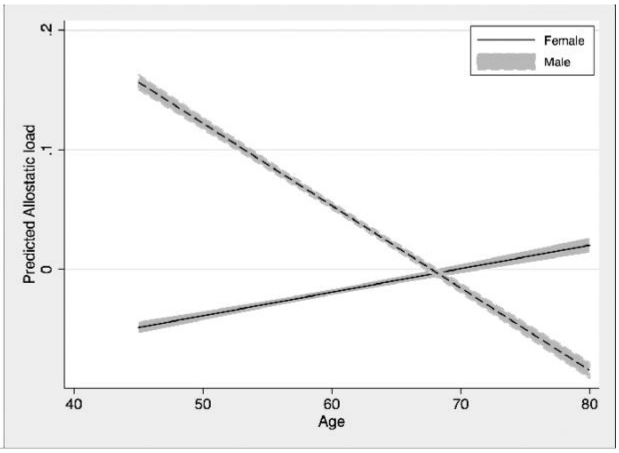

Supplement: S1 Fig — (PDF) [file pone.0315594.s004.pdf]
