# Supplementary material for: Sex differences in allostatic load trajectories among midlife and older adults: Evidence from the China health and retirement longitudinal study
Source: PLoS One. 2024 Dec 26;19(12):e0315594. doi: 10.1371/journal.pone.0315594 (PMC11670931; doi:10.1371/journal.pone.0315594)
Supplement: S2 Fig — (PDF) [file pone.0315594.s005.pdf]

S2 Fig: Predicted trajectories of allostatic load by gender:
socio-historical cohort before and after 1950: interaction models

(a) Weighted Model:
born between 1950-1970, 45-65 years old by 2015

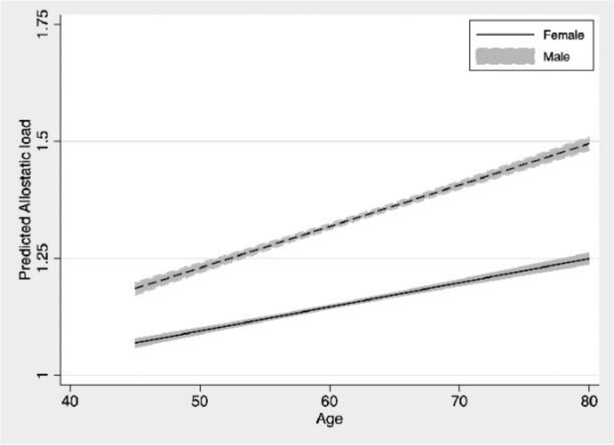

(b) Weighted Interaction Model:
born before 1950, 65 or older by 2015

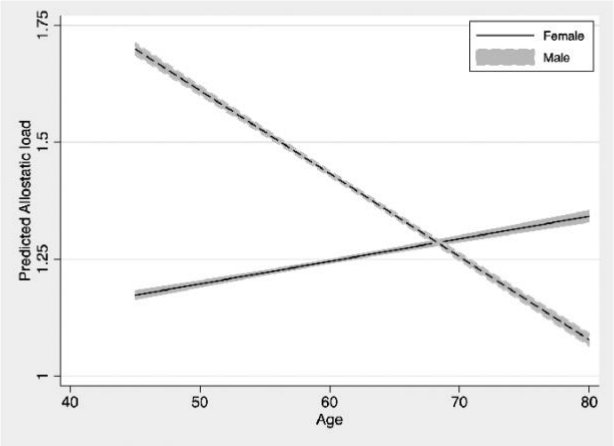

Note: S1 Fig b. is based on Model H, where AL is defined by z-score and IPW is used. The same to S2 Fig, where AL is defined by clinical cut-off points and top quartiles and IPW is used. As can be seen, IPW cannot provide a satisfactory adjustment to the age trajectories for older Chinese males and females: diverging AL trajectories (S1 Fig a.) and stronger interaction (S1 Fig b.). It is easy to understand the differences between MI and IPW because: Firstly, in IPW, missing values in AL were not included in the mixed-effect model, which decreased the observations significantly and led to statistical bias. Secondly, the ways to establish an IPW model vary in terms of what independent variables you chose. For instance, whether including the level of cognitive function in the IPW model will affect the scales of weights, hereby affecting the trajectories of AL. In contrast, MI will not decrease observations if not increase them. We believe MI is a more robust way to adjust for missingness in our study. Further, the aim of this study is to explore the sex difference in AL. IPW was introduced as a supplementary analysis to add evidence to the existence of a healthier cohort of males could be selected. To avoid introducing unnecessary complexity to the study, we decide not to include the comparisons and discussions of the differences between MI and IPW in the main texts of our study.
