# Supplementary material for: Sex differences in allostatic load trajectories among midlife and older adults: Evidence from the China health and retirement longitudinal study
Source: PLoS One. 2024 Dec 26;19(12):e0315594. doi: 10.1371/journal.pone.0315594 (PMC11670931; doi:10.1371/journal.pone.0315594)
Supplement: S3 Fig — (PDF) [file pone.0315594.s006.pdf]

S3 Fig: Predicted trajectories of allostatic load by gender: unbalanced data

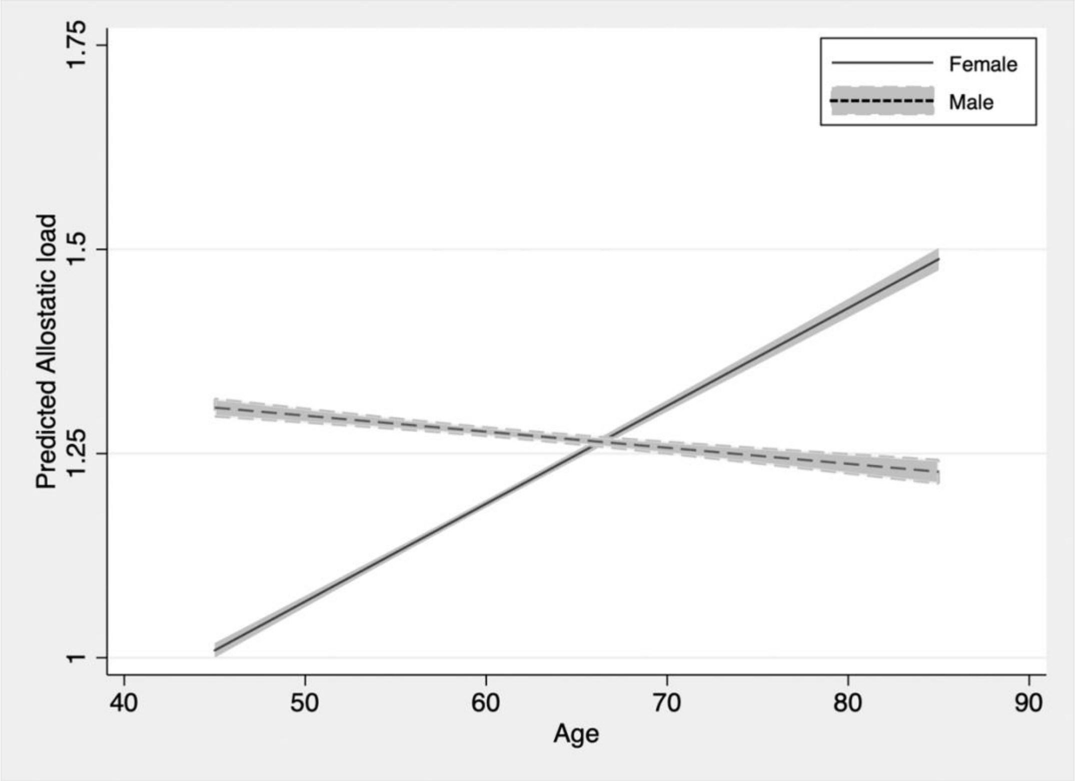

Note: AL is defined by clinical cut-off points, Multiple Imputation applied.
