# Supplementary material for: Sex differences in allostatic load trajectories among midlife and older adults: Evidence from the China health and retirement longitudinal study
Source: PLoS One. 2024 Dec 26;19(12):e0315594. doi: 10.1371/journal.pone.0315594 (PMC11670931; doi:10.1371/journal.pone.0315594)
Supplement: S1 Checklist — (PDF) [file pone.0315594.s007.pdf]

STROSA (version 2) Checklist

Item	Criterion	Availability of criteria Manuscript
**Title, abstract and keywords**		
1	Title and abstract	Page 1-2
2	Keywords	Page 2
**Introduction**		
3	Background and rationale	Page 3-5
4	Aim of the study	Page 5
**Methods**		
5	Study design	Page 6, first paragraph
6	Data source	Page 6, “data source and study sample”
7	Legal foundation	Page 7, “ethical consideration”
8	Data privacy	Page 7, “ethical consideration”
9	Data flow	Page 6, “data source and study sample”
10	Inclusion and exclusion criteria	Page 6, “data source and study sample”: we describe the inclusion criteria of a balanced dataset across two waves.<br>Page 7, “evaluation of allostatic load”: we describe the inclusion (biomarker availability) and exclusion (use of medication) criteria.
11	Unit of Analysis	Page 6, “data source and study sample”
12	Variables	Page 8, “covariates”.<br>Table S1 in supporting information.
13	Sample Size	Page 6, “data source and study sample”
14	Statistical analysis	Page 9-11. “Statistical Strategies”
**Results**		
15	Selection of study population	Page 12
16	Descriptive results	Page 12
17	Main results	Page 13-16
18	Further results	Page 13-16
**Discussion**		
19	Main results	Page 16-17
20	Internal validity and risk of bias	Page 18
21	Strengths and limitations	Page 19-20
22	Interpretation	Page 16-19
23	Transferability	Page 20-21
**Conclusion**		
24	Conclusion	Page 21
**Conflict of interest**		
25	Funding	There is no funding support this study.
26	Role of Data owner(s)	CHARLS is an open-source dataset. The owner (Peking University) has no influence on the implementation and presentation of the study.
27	Other conflict of interest	There were no other conflicts of interest; the evaluation was conducted independently.
